# CyberKnife Stereotactic Body Radiation Therapy as an Effective Treatment for Hepatocellular Carcinoma Patients With Decompensated Cirrhosis

**DOI:** 10.3389/fonc.2020.00100

**Published:** 2020-02-25

**Authors:** Jing Sun, Aimin Zhang, Wengang Li, Quan Wang, Jia Wang, Yuze Fan, Yingzhe Sun, Dong Li, Dan Zhang, Xuezhang Duan

**Affiliations:** Radiation Oncology Center, The Fifth Medical Center of PLA General Hospital (Beijing 302 Hospital), Beijing, China

**Keywords:** hepatocellular carcinoma, CyberKnife, stereotactic body radiation therapy, Child-Pugh B, Child-Pugh C

## Abstract

**Purpose:** The aim of our study was to evaluate the curative effect and safety of CyberKnife stereotactic body radiation therapy in treating decompensated cirrhosis hepatocellular carcinoma (HCC) patients.

**Methods:** From March 2011 to December 2015, 32 HCC patients who refused or were ineligible for other treatments were treated with CyberKnife stereotactic body radiation therapy. Among these patients, 17 were Child-Pugh score 7 (53.13%), 7 were Child-Pugh score 8 (21.87%), 4 were Child-Pugh score 9 (12.50%), and 4 were Child-Pugh score 10 (12.50%). A total dose of 45–54 Gy in 5–10 fractions was given according to the location of lesions.

**Results:** The median follow-up period was 30 months (8–46 months). By July 2019, the tumors were recurrent or metastasized in 17 patients. The overall survival rates of 1, 2, and 3 years were 84.4, 61.8, and 46.0%, respectively. After 1, 2, and 3 years, the local control rates were 92.9%. The progression-free survival rates of the 1, 2, and 3-year treatments were 73.8, 44.6, and 33.4%, respectively.

**Conclusions:** CyberKnife stereotactic body radiation therapy was an effective option for HCC patients with decompensated cirrhosis. The liver injury occurrence rate was acceptable in our study.

## Introduction

Hepatocellular carcinoma (HCC) is the sixth most common cancer worldwide and the fourth most common cause of cancer death (1). Most HCC patients occur with a history of chronic hepatitis, such as HBV, HCV infection, or alcoholic liver diseases. Therefore, when we choose the treatment modality for patients, sufficient hepatic capacity (2) is a prerequisite in HCC patients and needs to be evaluated accurately before treatment. To date, Child-Pugh classification (CP) is still widely accepted to evaluate liver function in clinical work, which provides a rough estimate of liver function by dividing patients into compensated (CP-A) or decompensated (CP-B, CP-C) cirrhosis. Furthermore, the CP score was shown to be associated with the patient prognosis (3–5).

For CP-A-HCC patients, the efficacy and safety of some treatments, such as resection, radiofrequency ablation (RFA), and percutaneous ethanol injection (PEI), have been reported in numerous studies based on the tumor stage. However, due to the higher risk of liver failure, treatment options are limited for patients with decompensated cirrhosis (especially Child-Pugh score more than 8). Liver transplantation (LT) is the optimal choice if these patients meet Milan criteria (6), but other treatments, such as resection and local ablation (including RFA, microwave ablation, etc.), could seldom be applied for these patients because of their poor liver function. Insufficient liver reserve also makes multidisciplinary team more cautious in choosing transcatheter arterial chemoembolization (TACE) or targeted therapy for fear of following liver failure.

Stereotactic body radiation therapy (SBRT) brings a satisfactory prognosis, especially for patients with inoperable or recurrent HCC (7, 8). CyberKnife SBRT (CK-SBRT) is a type of SBRT that has an absolute advantage in treating HCC by combining respiratory synchronous tracking and fiducial marker tracking, which could control the precision to be within 1 mm and achieve the precise therapy (9). The aim of our study was to evaluate the curative efficacy and safety of CK-SBRT in treating HCC patients with decompensated cirrhosis in our hospital.

## Materials and Methods

### Patient Selection

We conducted a retrospective review of 32 HCC patients with decompensated cirrhosis treated with CK-SBRT at The Fifth Medical Center of PLA General Hospital between March 2011 and December 2015. All patients met the criteria as follows: (a) the lesion was confined to the liver, (b) unfeasible or refusing to undergo other treatments, (c) residual normal liver volume ≥700 cc, (d) with decompensated cirrhosis (Child-Pugh B or C classification), and (e) without portal vein tumor thrombus. All patients voluntarily received CK-SBRT treatment and signed informed consent.

Twenty-four (75.00%) males and 8 (25.00%) females were enrolled in this study (Table 1). The median age was 55 years old (37–76 years old). Twenty-eight patients were chronic hepatitis B (87.50%), and four were chronic hepatitis C (12.50%). Seventeen patients had CP-7 scores (53.13%), seven had CP-8 scores (21.87%), four had CP-9 scores (12.50%), and four had CP-10 scores (12.50%). The tumor diameter was 2.80 cm (1.40–5.60 cm). Twenty-three patients (71.88%) were without previous treatment, and nine patients (28.12%) were previously underwent other methods, such as TACE and local ablation.

**Table 1:** Clinical and biochemical characteristics of patients enrolled in this study.

**Variables**	***n***
Gender	
Male	24 (75.00%)
Female	8 (25.00%)
Age (years)	
Median	55
Range	37–76
Type of Chronic hepatitis	
Hepatitis B	28 (87.50%)
Hepatitis C	4 (12.50%)
Child-Pugh score	
7	17 (53.13%)
8	7 (21.87%)
9	4 (12.50%)
10	4 (12.50%)
Maximum diameter of tumor (cm)	
Median	2.80
Range	1.40–5.60
AFP (ng/ml)	
Median	13.66
Range	1.42–23758
With previous treatment	
Yes	9 (28.12%)
No	23 (71.88%)

### Radiation Therapy

All enrolled patients were implanted three to four fiducials at 5 to 7 days prior to receiving SBRT, administered CyberKnife (Accuray, USA). The oncologist contoured the gross tumor volume (GTV). The planning target volume (PTV) was 3 mm expansion of GTV, which also avoided organs at risk. The prescribed dose delivered to the tumor was 45–54 Gy in 5–10 fractions. The isodose line of maximum dose was 72% (60–83%). The normal tissue dose was according to AAPM Task Group 101 (10).

The patients enrolled were re-evaluated every 3 months after treatment in the first year and then every 6 months until July 2019 or death.

### Toxicity Evaluation

The toxicity, based on the Toxicity Criteria of the Radiation Therapy Oncology Group and the European Organization for the Research and Treatment of Cancer (11), was graded during and after CK-SBRT.

### Radiation-Induced Liver Disease Assessment

Radiation-induced liver disease (RILD) was defined as the development of non-malignant ascites without disease progression and an anicteric elevation of alkaline phosphatase level by at least 2-fold. Non-classic RILD was defined as the development of jaundice and/or elevated serum transaminases (>5 × UL) within 3 months of completion of RT in patients with underlying chronic hepatic disease (cirrhosis or viral hepatitis) (12).

### Statistical Analysis

OS was defined as the period between date of CK-SBRT beginning and the date of final follow-up or patient's death. LC was defined as the period between the date of CK-SBRT and the date of the progression of lesion treated or patient's death. PFS was defined as the period between the date of CK-SBRT and the date of disease progression or patient's death.

The Kaplan–Meier method was used for estimating OS, LC, and PFS. Log-rank test was applied to compare OS, LC, and PFS between groups. All statistical analyses were performed using Statistical Package for the Social Sciences software (SPSS ver. 22.0, IBM Corp., Armonk, NY) and Software for Statistics and Data Science (STATA ver. 15.0, STATA Corp., College Station, TX, USA).

### Follow-Up Study

After treatment with CK-SBRT, patients were reviewed every 3 months within the first year and thereafter every 6 months until July 2019. The follow-up included laboratory results and abdominal CT/MRI, lung CT, brain CT/MRI, and PET-CT examination if necessary. Our retrospective study was conducted in observing OS, PFS, and LC.

## Results

### Tumor Recurrence, Treatment, and Survival Outcomes

Median follow-up period was 30 months (8–46 months). By July 2019, the tumors were relapsed or metastasized in 17 patients. Among them, 15 had metastases in the liver, 1 in a lymph node, and 1 in the brain. When recurrence/metastases were confirmed, second-line treatment was individualized according to the number and location of the recurrent tumor and liver function status and in consideration of patient preference. Therapeutic options included repeated SBRT (5 patients) and conservative treatment (12 patients).

By July 2019, 20 patients died, including 9 patients who died of hepatic failure, 6 with upper gastrointestinal bleeding, 2 with sepsis and septic shock, 1 with pulmonary failure, and 2 with hepatic encephalopathy (Table 2).

**Table 2:** The details of patients and the parameters of CK-SBRT.

**Number of patients**	**Maximum diameter of tumor (cm)**	**Total prescribed dose (Gy)**	**Residual normal liver volume (cc)**	**D 700 (Gy)**	**Child-Pugh score**	**Total bilirubin (μmol/L)**	**Albumin (g/L)**	**Relapse time (months)**	**Follow-up period (months)**	**Living/dead**	**Cause of death**
1	4.90	49	1,055	7.41	7	25.50	33	–	8	Dead	Hepatic failure
2	5.00	54	1,050	4.00	10	55.80	27	7	10	Dead	Hepatic failure
3	2.60	54	965	6.10	9	45.50	29	–	35	Dead	Upper gastrointestinal bleeding
4	3.80	50	1,128	2.30	7	44.10	36	7	10	Dead	Hepatic failure
5	4.20	50	845	8.70	7	10.90	39	14	37	Dead	Hepatic encephalopathy
6	2.20	50	736	3.30	8	17.50	33	–	28	Dead	Upper gastrointestinal bleeding
7	1.50	49	1,109	5.20	10	52.10	26	21	38	Dead	Hepatic failure
8	2.70	50	1,547	5.50	9	45.20	25	–	23	Dead	Upper gastrointestinal bleeding
9	4.40	54	1,266	11.20	8	14.40	32	20	33	Dead	Hepatic failure
10	2.90	50	1,517	11.50	8	27.90	31	41	41	Dead	Hepatic failure
11	1.70	50	918	2.30	7	28.30	30	15	37	Living	
12	5.50	45	1,055	7.41	7	22.00	26	–	8	Dead	Hepatic encephalopathy
13	1.40	54	1,212	4.00	7	28.70	28	–	37	Dead	Hepatic failure
14	1.60	50	735	6.46	7	34.20	31	15	30	Dead	Septic shock
15	5.60	50	724	6.10	7	42.80	37	12	31	Living	
16	3.80	54	1456	11.80	9	56.00	31	–	24	Dead	Hepatic failure
17	3.30	54	1,027	4.50	10	45.40	25	8	10	Dead	Upper gastrointestinal bleeding
18	3.50	50	1171	6.90	9	35.20	26	3	17	Dead	Pulmonary failure
19	2.20	49	1,029	5.27	7	60.30	35	–	31	Living	
20	4.90	54	711	9.60	10	37.50	26	34	46	Living	
21	1.80	54	1337	8.94	8	9.40	19	24	27	Living	
22	2.00	49	932	4.70	7	16.40	35	–	15	Living	
23	2.50	54	717	2.00	7	39.50	38	–	13	Dead	Hepatic failure
24	1.80	50	804	5.40	7	22.80	29	14	17	Dead	Septic shock
25	1.90	50	1,054	7.54	8	34.90	29	12	14	Dead	Upper gastrointestinal bleeding
26	4.70	49	1,196	7.70	8	32.20	27	–	45	Living	
27	1.80	50	1,397	8.40	7	24.00	32	–	41	Living	
28	3.00	54	716	4.30	7	22.60	25	8	19	Dead	Upper gastrointestinal bleeding
29	1.60	50	1,483	2.00	7	25.20	34	12	38	Living	
30	1.70	54	1,278	1.87	7	27.90	32	–	33	Living	
31	5.00	54	828	0.90	8	36.80	30	–	32	Living	
32	3.20	50	1,046	4.10	7	28.10	34	–	29	Living	

*D700, dose received by 700 cc (cm^3^) of residual normal liver*.

**Figure 1:**
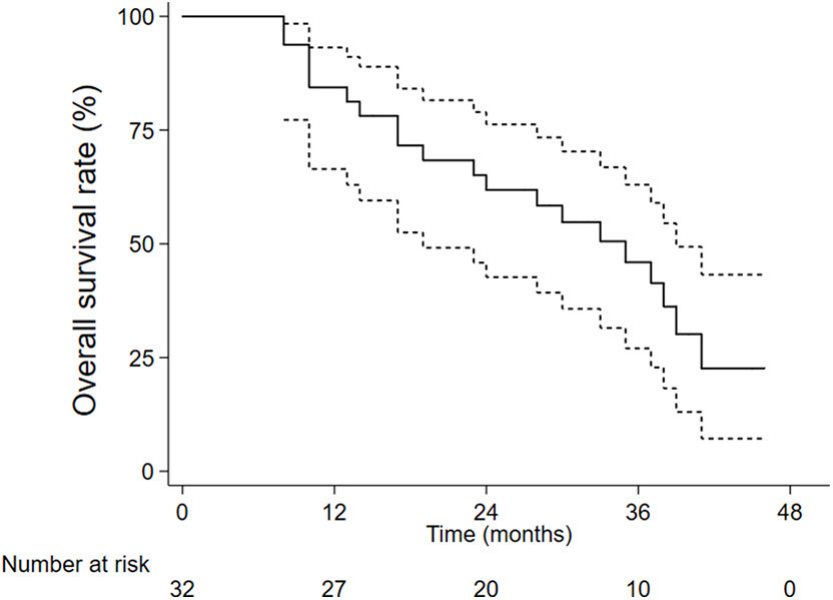
The overall survival rates.

**Figure 2:**
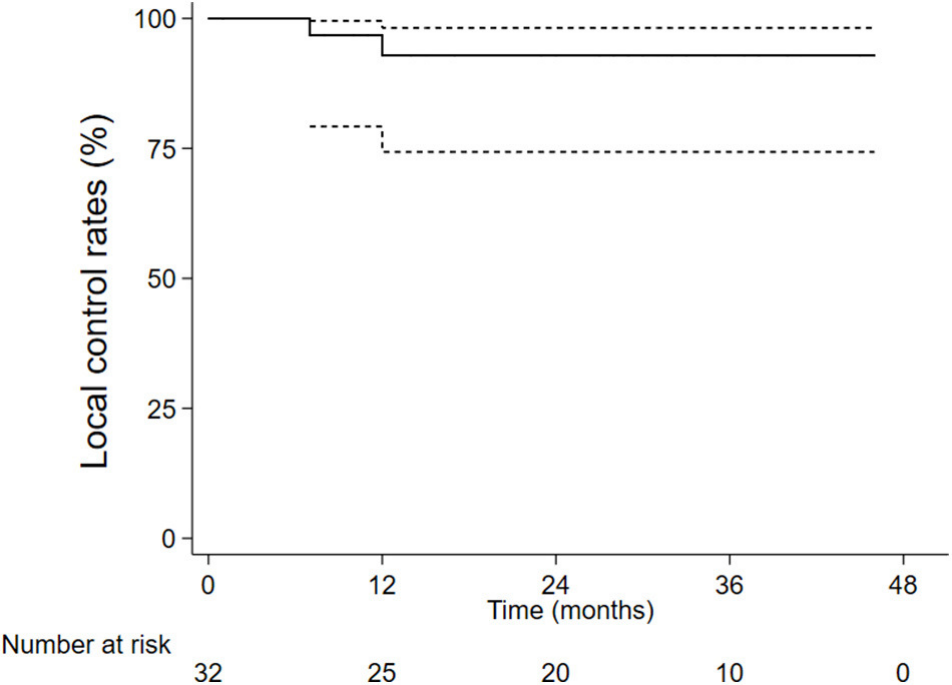
The local control rates.

**Figure 3:**
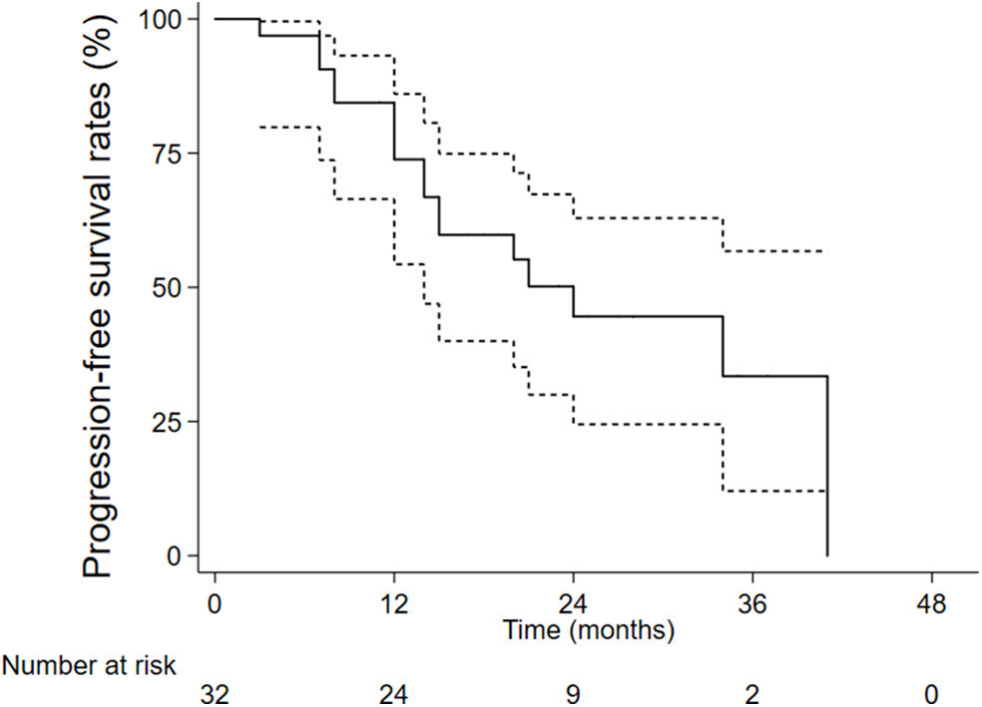
The progression-free survival rates.

The 1-, 2-, and 3-year overall survival rates were 84.4, 61.8, and 46.0%, respectively (Figure 1). Local control rates of 1-, 2-, and 3-year were maintained at the same level of 92.9, 92.9, and 92.9% (Figure 2). Progression-free survival rates of 1, 2, and 3 years were 73.8, 44.6, and 33.4%, respectively (Figure 3).

**Figure 4:**
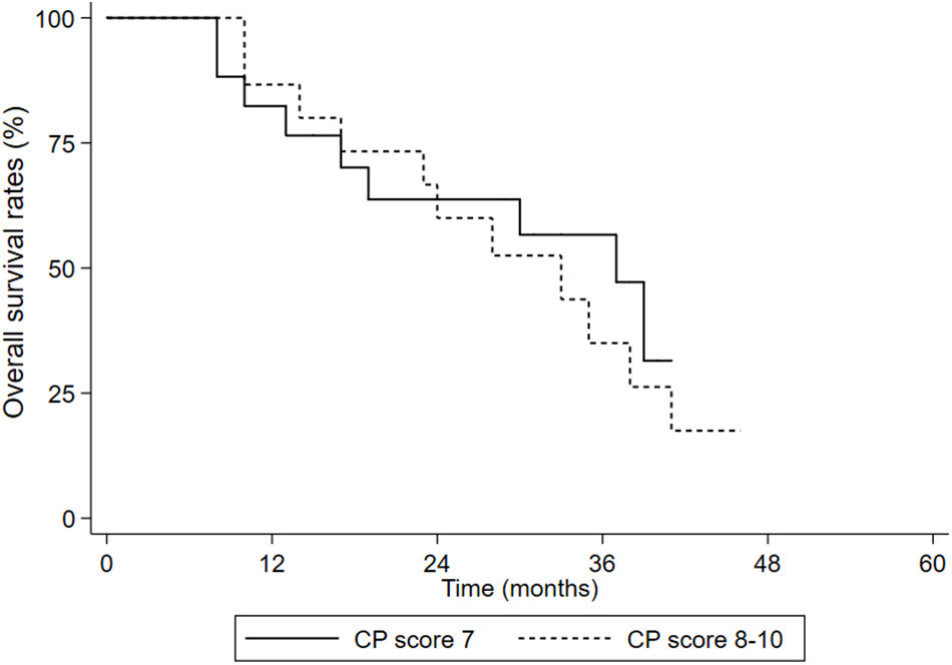
OS in the CP score 7 group and CP score 8–10 groups.

**Figure 5:**
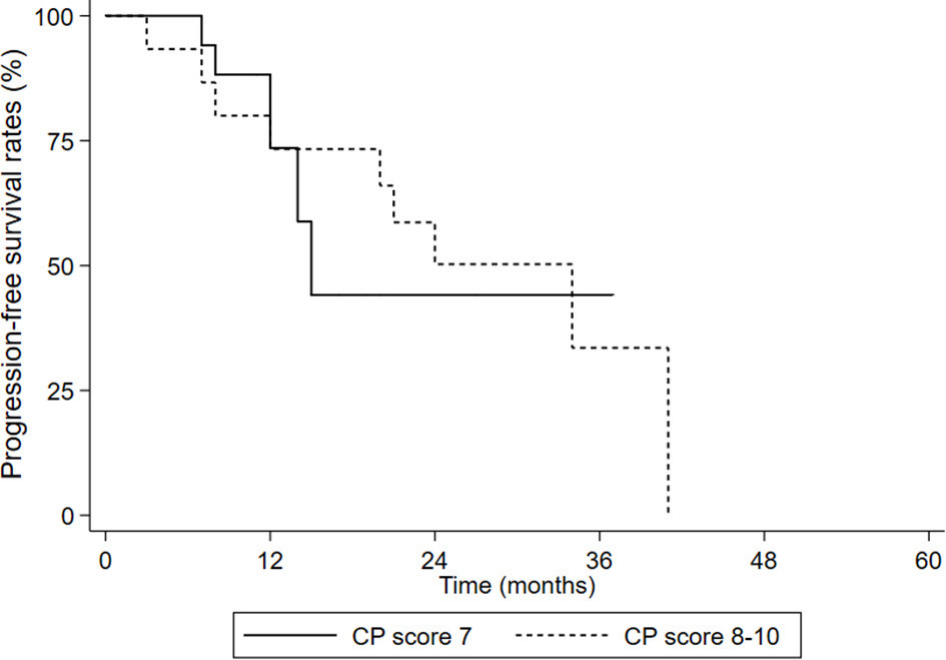
PFS in the CP score 7 group and CP score 8–10 groups.

We divided the patients into two groups: CP 7 scores group and CP 8–10 scores group. OS and PFS in the two groups were all not statistically different (OS: *p*= 0.614, Figure 4; PFS: *p* = 0.561, Figure 5).

### Toxicity Outcomes

CK-SBRT was completed in all 32 patients. Sixteen patients had Grade 1–2 acute toxicity reactions, including fatigue, abdominal pain, anorexia, and vomiting, which could be relieved gradually by corresponding treatment. No patients had Grade ≥3 acute toxicity reactions.

### Liver Toxicity

**Figure 6:**
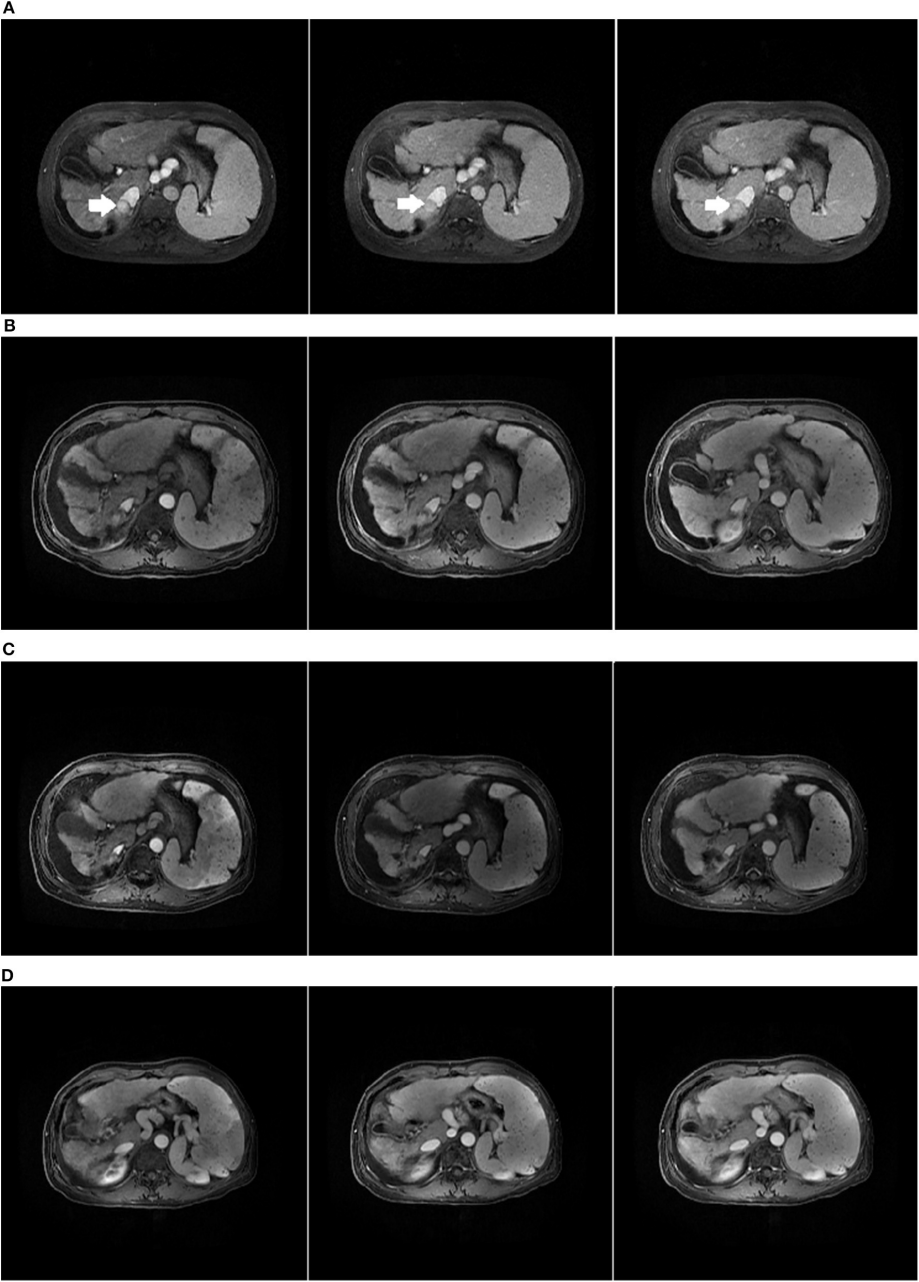
Child-Pugh C HCC patient who received CK-SBRT assessed by MRI. **(A)** The initial abdominal MRI scan with the primary HCC indicated by the arrow. **(B)** MRI scan of 3 months after SBRT. **(C)** MRI scan of 24 months after SBRT. The lesion in the liver disappeared. **(D)** MRI scan of 42 months after SBRT.

Among the enrolled patients, four patients were diagnosed with RILD, and they were all relieved after drug treatment. Among the four CP-C patients in our study, one patient was still alive, and the survival time was 46 months. Before CK-SBRT, the patient belonged to Child-Pugh score C10 (total bilirubin = 37.5 μmol/L, albumin = 26 g/L, large-volume ascites). Until July 2019, there was no recurrence or metastasis in this patient. The result in July 2019 showed that the patient was a Child-Pugh score B9 (total bilirubin = 80.5 μmol/L, albumin = 30 g/L, small-volume ascites). The patient medical images are shown in Figure 6. We considered that CP-C patients who did not receive LT should also receive active treatment to prolong their survival.

## Discussion

Generally, based on chronic hepatitis B or C virus infection in China, HCC patient treatments were limited not only by advanced or end stage at diagnosis but also by poor liver function. Previous studies (13–15) have shown that poor Child-Pugh scores affect not only choice of treatment but also the prognosis of HCC patients. Decompensated liver cirrhosis patients may be more at risk of dying from cirrhosis complications than from tumor dissemination.

As an option, LT could cure not only the tumor but also liver cirrhosis, thus reducing the risk of liver failure. Therefore, LT is a primary choice for CP-B-HCC patients who meet the Milan criteria. In addition to LT, different types of hepatectomy were also the choices for the patients who had enough liver reserve. Shintaro Kuroda (16) compared the OS of CP-B-HCC patients between hepatectomy and transplantation groups. Their results showed that the OS at 1 and 3 years after LT was 87.0 and 78.3%, respectively. Moreover, after hepatectomy, the OS at 1 and 3 years was 81.9 and 52.9%, respectively, which is similar to 84.4 and 61.8% in our study.

However, LT is limited due to a lack of donors and strict criteria. In addition, local ablation, TACE, and targeted therapy were selectively applied to part of CP-B but not CP-C HCC patients. However, each of these treatments has limitations. Alessandro Granito (17) summarized the limits of CP-B-HCC patient treatment applicability. Among early stage patients, local ablation was not suitable for those with ascites or coagulopathy or both (frequent in CP-B). Among middle stage patients, TACE was only applied for those who were CP B7 and without ascites (EASL guidelines recommend). Moreover, sorafenib was not suggested in advanced stage CP-B patients. Among these methods, local ablation is a radical therapy. Yang (18) reported the post-RFA OS of HCC patients. The 1-year and 3-year OS with CP-B liver function were 82.5 and 46.8%, respectively, whereas the OS was 28.6 and 14.3% for CP-C, respectively. Our study included both CP-B and CP-C patients, but our 1-year OS was similar to the CP-B level in his study. Surprisingly, our 3-year OS was higher than the CP-B patients reported above. In our study, SBRT had a similar effect as RFA or even better.

Compared with CP-B patients, CP-C patient treatment options were more limited. The European Organization for Research and Treatment of Cancer (EASL-EORTC) (13) guideline and the American Association for the study of Liver Disease (AASLD) (19) guidelines all recommend LT for patients meeting the Milan criteria and best supportive care for patients exceeding the criteria. For these patients, more treatment methods are worth exploring. Masatoshi Kudo (20) reported a retrospective multicenter study of 436 HCC patients with Child-Pugh C cirrhosis. Their results showed that liver non-transplant treatments (i.e., TACE, RFA, PEI therapy, and hepatic arterial infusion chemotherapy) confer survival benefit in these patients. Therefore, despite the poor prognosis of CP-C disease, the treatment might still prolong their survival.

SBRT has been applied in HCC patients since 20 years ago (21). In recent years, an increasing number of studies have shown that SBRT has achieved a satisfactory effect on HCC patients. The incidence of liver injury has decreased significantly compared with conventional radiotherapy (CRT). In addition, similar to CRT, the risk of RILD was also higher in CP-B patients than in CP-A (3–5) patients (22). To reduce liver injury, research teams always limited Child-Pugh score >8 as a contraindication. Therefore, few studies included CP-B patients with score >8 and CP-C patients.

Culleton (15) conducted studies in this field. He reported outcomes in patients with CP-B/C HCC treated with SBRT (median dose 30 Gy in 6 fractions). The 12-month survival rate was 32.3%, and the median survival of Child-Pugh B7 patients was 9.9 months vs. 2.8 months for CP score ≥8. The proportion of patients with CP-B7 (69%) in Culleton's study was higher than that in our study, but the OS was significantly lower than ours. A possible explanation for this finding might be that 76% of the patients in their study had portal vein thrombus, but none had portal vein thrombus, and the diameter of the tumors (median: 2.80 cm) in our enrolled patients was obviously smaller than that of their research group. Previous studies proved that tumor volume (23–26) and portal vein thrombus were all negative factors influencing survival. Therefore, compared with his study, our study focused more on early stage HCC patients. In addition, there was only one CP-C patient in their study, but four CP-C patients in our study, among whom one patient was still alive. This result may provide new options for the treatment of CP-C patients.

Although the prescribed dose in his study was lower than ours, the liver injury occurrence rate (17%) was higher than that in our study (12.5%). It is worth noting that we did not lower the liver tolerance dose standard in the AAPM TG101 report for poor liver function, but the RILD rate was acceptable. Compared with the previous study, in addition to differences between the enrolled patient criteria, he adopted 3D-CRT, IMRT, and VMAT to implement SBRT. CK-SBRT adopted fiducial marker tracking combined with dynamic respiration tracking, which improved accuracy by non-coplanar irradiation and better protected residual normal liver. However, fiducial marker implantation was an invasive operation that may increase risk of bleeding and infection, especially for decompensated cirrhosis patients who were usually with thrombocytopenia and ascites. Before implantation, recombinant human thrombopoietin (rh-TPO) was usually given to the patients with thrombocytopenia until the peripheral platelet count reached beyond 50 × 10^9^/L. For patients with ascites, ascites lab tests including WBC count and bacteria culture were analyzed to exclude spontaneous bacteria peritonitis (SBP). In addition, the operation protocol was standardized and strictly controlled during every step to ensure the sterile operation. There was no case of bleeding and infection in this study. We considered that these risks above could be prevented or handled well.

Moreover, the OS and PFS had no statistical differences in the CP score 7 group and the CP score 8–10 group. For HCC patients who received radiation therapy, RILD was a serious adverse reaction. Previous studies showed CP score was an influence factor of RILD, which may affect the prognosis of the patients. However, none of patients died from RILD in our study, and the result indicated the main causes of death were complications of cirrhosis itself. By reviewing the planning parameters of enrolled patients, we found that the residual normal liver volume was large enough even in patients with a CP score 8–10, which may maintain normal liver function level to withstand radiation injury. Therefore, we considered that we should pay more attention to tumor size and residual normal liver in SBRT of decompensated cirrhosis HCC patients.

However, our sample size was too small, and the result might be influenced by some random factors. A large sample of prospective studies of SBRT in HCC patients with decompensated cirrhosis is urgently needed to add evidence for this treatment. However, because there are only a few institutions to carry out the treatment of CP > 8 score patients, multicenter studies may be the only way to achieve this goal.

## Conclusion

CK-SBRT was an effective option for HCC patients with decompensated cirrhosis. The liver injury occurrence rate was acceptable in our study. More large-sample size studies about prognosis and influencing factors are worth exploring.

## Data Availability Statement

The datasets used and analyzed during this study are available from the corresponding author on reasonable request.

## Ethics Statement

This study protocol was permitted by the Institutional Review Board of 302 Hospital of PLA (People's Liberation Army) and was treated in accordance with the Declaration of Helsinki. All patients gave written consent for getting treated with CK-SBRT.

## Author Contributions

JS and AZ: design of the research and drafted the manuscript. WL, QW, and JW: analysis and interpretation of data. JS, YF, and YS: acquisition of data. DL and DZ: follow-up content. XD: revised and approved final version of manuscript.

### Conflict of Interest

The authors declare that the research was conducted in the absence of any commercial or financial relationships that could be construed as a potential conflict of interest.
